# A stack LSTM structure for decoding continuous force from local field potential signal of primary motor cortex (M1)

**DOI:** 10.1186/s12859-020-03953-0

**Published:** 2021-01-22

**Authors:** Mehrdad Kashefi, Mohammad Reza Daliri

**Affiliations:** Neuroscience and Neuroengineering Research Lab., Biomedical Engineering Department, School of Electrical Engineering, Iran University of Science and Technology (IUST), Tehran, Iran

**Keywords:** LFP, Force decoding, LSTM, BCI

## Abstract

**Background:**

Brain Computer Interfaces (BCIs) translate the activity of the nervous system to a control signal which is interpretable for an external device. Using continuous motor BCIs, the user will be able to control a robotic arm or a disabled limb continuously. In addition to decoding the target position, accurate decoding of force amplitude is essential for designing BCI systems capable of performing fine movements like grasping. In this study, we proposed a stack Long Short-Term Memory (LSTM) neural network which was able to accurately predict the force amplitude applied by three freely moving rats using their Local Field Potential (LFP) signal.

**Results:**

The performance of the network was compared with the Partial Least Square (PLS) method. The average coefficient of correlation (r) for three rats were 0.67 in PLS and 0.73 in LSTM based network and the coefficient of determination ($R^{2}$) were 0.45 and 0.54 for PLS and LSTM based network, respectively. The network was able to accurately decode the force values without explicitly using time lags in the input features. Additionally, the proposed method was able to predict zero-force values very accurately due to benefiting from an output nonlinearity.

**Conclusion:**

The proposed stack LSTM structure was able to predict applied force from the LFP signal accurately. In addition to higher accuracy, these results were achieved without explicitly using time lags in input features which can lead to more accurate and faster BCI systems.

## Background

The advent of Brain Computer Interfaces (BCIs) holds the promise to restore movement to disabled limbs or control an artificial effector [1, 2]. Various signal recording methods with different level of invasiveness had been used in BCI systems; despite the high level of invasiveness, the intracortical recordings have the highest movement-related information and signal to noise ratio [3]. The intracortical neural data, usually spike time series or firing rate of neurons, are used to continuously control the movement of the artificial effector or stimulate the disabled limb. However, recording spikes over a long period can be challenging. The number of received spikes decreases over time and reduces the functionality of the BCI system. Also, spikes are recorded at high sampling frequency, which increases the complexity and cost of recording devices. Therefore, Local Field Potentials (LFP) are used as a more stable and simpler source of information [4]. LFPs are low-frequency voltage fluctuations that it is believed to be related to activities of postsynaptic currents nearby the recording electrodes [5]. Many studies have shown that movement-related parameters can be decoded using spectral features from multi-channel LFP signal [6, 7]. For instance, the continuous position of the hand in both 2D and 3D space was decoded using LFP signals recorded from motor cortex area M1 [8]. However, many daily activities, like grasping, require controlling the precise value of force applied to an object. Therefore, it would be useful to be able to decode force-related information from brain signals. Some studies [9–11] used the ECoG signal, recorded from movement-related areas, to predict force amplitude. Among the few studies regarding force decoding using LFP, Milecovic et al. [11] used 100 LFP channels to decode accurate force applied to each finger during a grasping task. Khorasani et al. used merely 16 LFP channels (the same data used in this study) to decode force value applied by a freely moving rat [12].

All neural data, including LFPs, are highly dynamic, nonlinear and have low signal to noise ratio. These challenging characteristics of neural data compelled researcher to use various signal processing and machine learning approaches to achieve higher performance in decoding movement-related parameters from neural data. For instance, Khorasani et al*.* proposed a novel adaptive artifact removal technique for enhancing signal quality to achieve higher performance in BCI [13]. Foodeh et al*.* introduced the minimum noise estimate (MNE) filter for removing artifacts from the recorded neural signals [14]. Marathe et al*.* modified common spatial patterns (CSP) technique for feature extraction in continuous BCI systems [15]. Benz et al*.* proposed a novel feature extraction schema via connectivity analysis in continuous BCIs [16]. Zhuang et al*.* considered the dynamic characteristics of BCI’s output by applying a Kalman filter as the decoder [8]. Zheng Li et al*.,* and Simin Li et al*.,* modified the unscented Kalman filter to non-linearly estimate movement-related parameters [17, 18]. Shimoda et al*.* used partial least squares (PLS) regression for decoding 3-D hand trajectories for dealing with high-dimensionality of features space [19]. van Gerven et al*.* introduced sparse orthonormalized PLS as an extension to the ordinary PLS, which can simultaneously perform feature selection and regression [20].

Among all the complexities of designing a BCI decoding system, handling large dimensionality of the feature space, learning the intrinsic dynamic of data, and finding the possible nonlinear mapping between neural data and the target movement parameters seems to be necessary for a successful BCI decoding model. In most of the cases, however, the proposed methods fail to address these challenges simultaneously. For instance, many regression methods, like the family of linear methods, can neither find the intrinsic dynamic of data, nor the nonlinear mapping between the input and output. Thus, to solve the problem of intrinsic dynamics, multiple prior time samples of data are added to features. Nonetheless, the concern regarding nonlinear mapping persists, and adding previous time samples also increases the dimensionality of feature space. Therefore, it is natural to think of a nonlinear method in which the previous relevant features are automatically used for predicting the current target value. Recurrent neural networks have the aforementioned characteristics.

Recurrent neural networks (RNNs) can learn the intrinsic dynamic of data. However, due to gradient vanishing, the information from the past samples cannot easily reach the current sample [21]. To solve this problem, new structures, including LSTM (Long Short-Term Memory) and GRU (Gated Recurrent Units), were introduced [22]. Long short-term memory networks have an extra path for transferring relevant information from the previous sample to more recent ones. Unlike classical recurrent neural networks, LSTM is more robust to gradient vanishing and was considerably successful in natural language processing and time series prediction. It is expected that these networks can also be able to learn relevant information in neural data. For instance, Belo et al. [23] used A GRU based structure for synthesizing multiple biological signals including Electrocardiogram (ECG) and Electromyogram (EMG) with the purpose of denoising, classification and generating (reproduction) of EMG and ECG signals. Also, in another case, LSTM network was used to predict hand kinematics [24]. Unfortunately, unlike natural language processing, there are few samples of data in BCI related datasets. Therefore, LSTMs are prone to be overfitted to training data. Beside regularization, Dropout layers proved to be useful in controlling overfitting problem [25]. In this study, an LSTM based neural network is used to decode the force amplitude from the spectral features of LFP data without directly using time lags in features. The results are compared with the Partial Least Square (PLS) method.

## Results

The network is evaluated using seven-fold cross-validation. In this method, data is divided into 7 partitions, and each time, 6 partitions are used as train and the remaining partition as test data. This process is repeated 7 times, and the final performance is the average performance in all the 7 folds. The same train and test data are used in PLS evaluation. The hyper-parameters of the LSTM network and the number of components for PLS were optimized as explained in the method section for each fold.

### Prediction accuracy

The correlation coefficient (r) and coefficient of determination ($R^{2}$) are reported for each rat in all the iteration in Tables 1 and 2, respectively. In both Tables 1 and 2, the highest values are italic. In terms of correlation, the mean value for all 7-folds for rat1, rat 2 and rat 3 are 0.7 ± 0.05, 0.6 ± 0.06, 0.71 ± 0.02 for PLS with 10 time lags and 0.74 ± 0.05, 0.70 ± 0.05 and 0.75 ± 0.03 for LSTM Network respectively. The statistical significance of the results was tested using the Wilcoxon signed-rank test p < 0.01. In terms of coefficient of determination ($R^{2}$), the mean value of 7 folds for rat 1, rat 2 and rat 3 are 0.44 ± 0.06, 0.43 ± 0.1 and 0.49 ± 0.05 for PLS and 0.52 ± 0.08, 0.49 ± 0.09 and 0.54 ± 0.06 for LSTM Network respectively with statistical significance p < 0.05 (Wilcoxon signed-rank test). These results indicate that the LSTM Network was able to predict the force value more accurately. For additional evaluation of prediction accuracy, we also evaluated the network with 7-times 7-folds cross-validation, and the significance of the results was evaluated for each rat (see Additional file 1).

**Table 1:** Correlation coefficient of LSTM network and PLS (10 lags), for all rats and 7 folds

*r*	Rat 1		Rat 2		Rat 3	
	*PLS*	*LSTM*	*PLS*	*LSTM*	*PLS*	*LSTM*
Fold 1	0.74	0.74	0.75	*0.77*	0.70	0.76
Fold 2	0.65	0.71	0.76	0.76	0.76	0.79
Fold 3	0.63	0.68	0.70	0.75	0.68	0.74
Fold 4	0.69	0.80	0.67	0.64	0.73	0.77
Fold 5	0.68	*0.82*	0.57	0.62	0.69	0.69
Fold 6	0.79	0.71	0.76	0.70	0.71	*0.80*
Fold 7	0.74	0.78	0.67	0.72	0.72	0.74
Average	0.7 ± 0.05	0.74 ± 0.05	0.69 ± 0.06	0.70 ± 0.05	*0.71* ± *0.02*	0.75 ± 0.03

**Table 2:** Coefficient of determination for LSTM network and PLS (10 lags), for all rats and 7 folds

*R*^2^	Rat 1		Rat 2		Rat 3	
	*PLS*	*LSTM*	*PLS*	*LSTM*	*PLS*	*LSTM*
Fold 1	0.50	0.54	0.36	*0.59*	0.48	0.55
Fold 2	0.38	0.51	0.57	0.57	0.57	*0.62*
Fold 3	0.39	0.44	0.49	0.49	0.41	0.48
Fold 4	0.43	0.46	0.42	0.42	0.53	0.59
Fold 5	0.38	*0.68*	0.29	0.34	0.46	0.45
Fold 6	0.53	0.45	0.54	0.57	0.50	*0.62*
Fold 7	0.48	0.58	0.35	0.49	0.50	0.52
Average	0.44 ± 0.06	0.52 ± 0.08	0.43 ± 0.1	0.49 ± 0.09	0.49 ± 0.05	0.54 ± 0.06

**Fig. 1:**
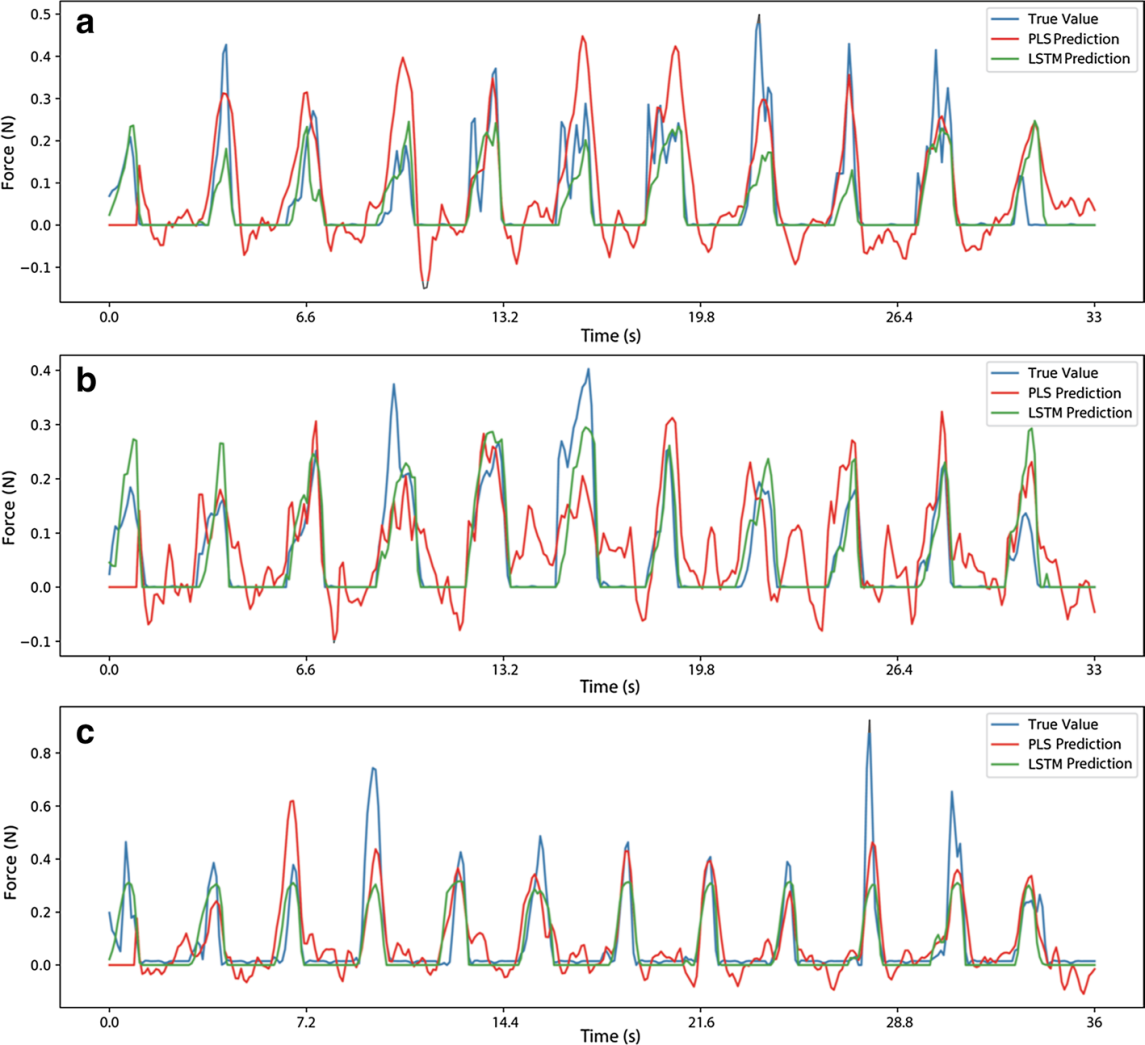
Predicted force value for LSTM and PLS for three rats. The blue line indicates the true force value, the red and the green lines are PLS and LSTM predictions respectively. **a** Rat 1: r = 0.82 and ${\text{R}}^{2} = 0.68$, **b** Rat 2: r = 0.77 and ${\text{R}}^{2} = 0.59$, **c** Rat 3: r = 0.80 and ${\text{R}}^{2} = 0.62$

To have a better visualization of the predictions, three predictions with the highest ($R^{2}$) values are plotted in Fig. 1. The force value predicted by LSTM and PLS, and the observed force values are compared. The blue line indicates the true force value, the red and green lines are predictions made by PLS and LSTM, respectively. For each rat, the folds with the highest decoding accuracy are plotted. As can be seen in Fig. 1, LSTM was able to follow the true force value more accurately.

### CAR filtering

In order to evaluate the effect of CAR filter, force values were predicted once with applying CAR filter on the LFP signal and once without CAR filter. The effect of CAR filter was investigated for both PLS and LSTM Network and all rats. The results are shown in Tables 3 and 4. The results show that using CAR filter improves the prediction accuracy for both PLS and LSTM Network. This improvement was significant for both PLS and LSTM (p < 0.05 Wilcoxon signed-rank test for all rats and all folds combined).

**Table 3:** Correlation coefficient of LSTM network and PLS, with and without CAR filter

r	Rat 1		Rat 2		Rat 3	
	*PLS*	*LSTM*	*PLS*	*LSTM*	*PLS*	*LSTM*
CAR filter	0.7 ± 0.05	0.74 ± 0.05	0.69 ± 0.06	0.70 ± 0.05	0.71 ± 0.02	0.75 ± 0.03
No CAR filter	0.6 ± 0.02	0.70 ± 0.04	0.58 ± 0.04	0.68 ± 0.04	0.68 ± 0.02	0.71 ± 0.01

**Table 4:** Coefficient of determination for LSTM network and PLS, with and without CAR filter

*R*^2^	Rat 1		Rat 2		Rat 3	
	*PLS*	*LSTM*	*PLS*	*LSTM*	*PLS*	*LSTM*
CAR filter	0.44 ± 0.06	0.52 ± 0.08	0.43 ± 0.1	0.49 ± 0.09	0.49 ± 0.05	0.54 ± 0.06
No CAR filter	0.39 ± 0.06	0.42 ± 0.07	0.40 ± 0.2	0.43 ± 0.07	0.42 ± 0.03	0.51 ± 0.04

### Contribution of time lags in PLS prediction

**Fig. 2:**
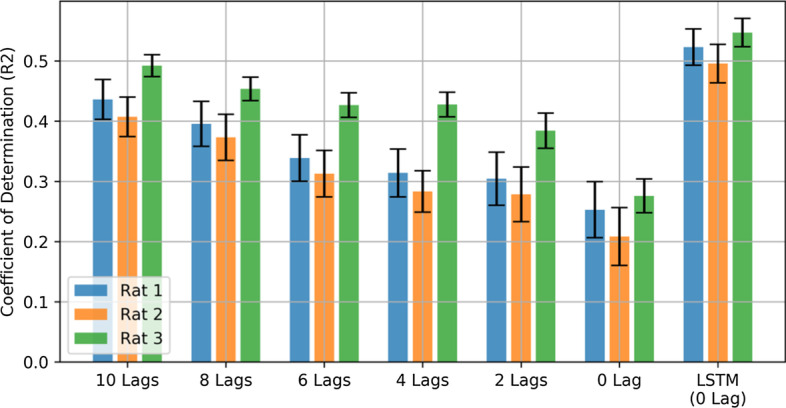
Coefficient of Determination $({\text{R}}^{2})$ of PLS predictions for the different number of time lags. Values from 10 time lags prediction differ significantly from values with no time lag (p < 0.01 Wilcoxon signed-rank test)

In order to investigate the effect of time lags in PLS prediction, PLS was evaluated with different number of time lag. The best results were obtained by considering 10 time lags. In Fig. 2, the coefficient of determination ($R^{2}$) for PLS predictions with a different number of time lags is illustrated. For rat 1 ($R^{2}$) value of prediction significantly decreases from 0.44 ± 0.06 to 0.25 ± 0.13; the same pattern is observed for the other rats. In summary, values from 10 time lags prediction differ significantly from values with no time lag (p < 0.01 Wilcoxon signed-rank test), which indicates that there is relevant information in previous samples.

### Contribution of frequency bands

**Fig. 3:**
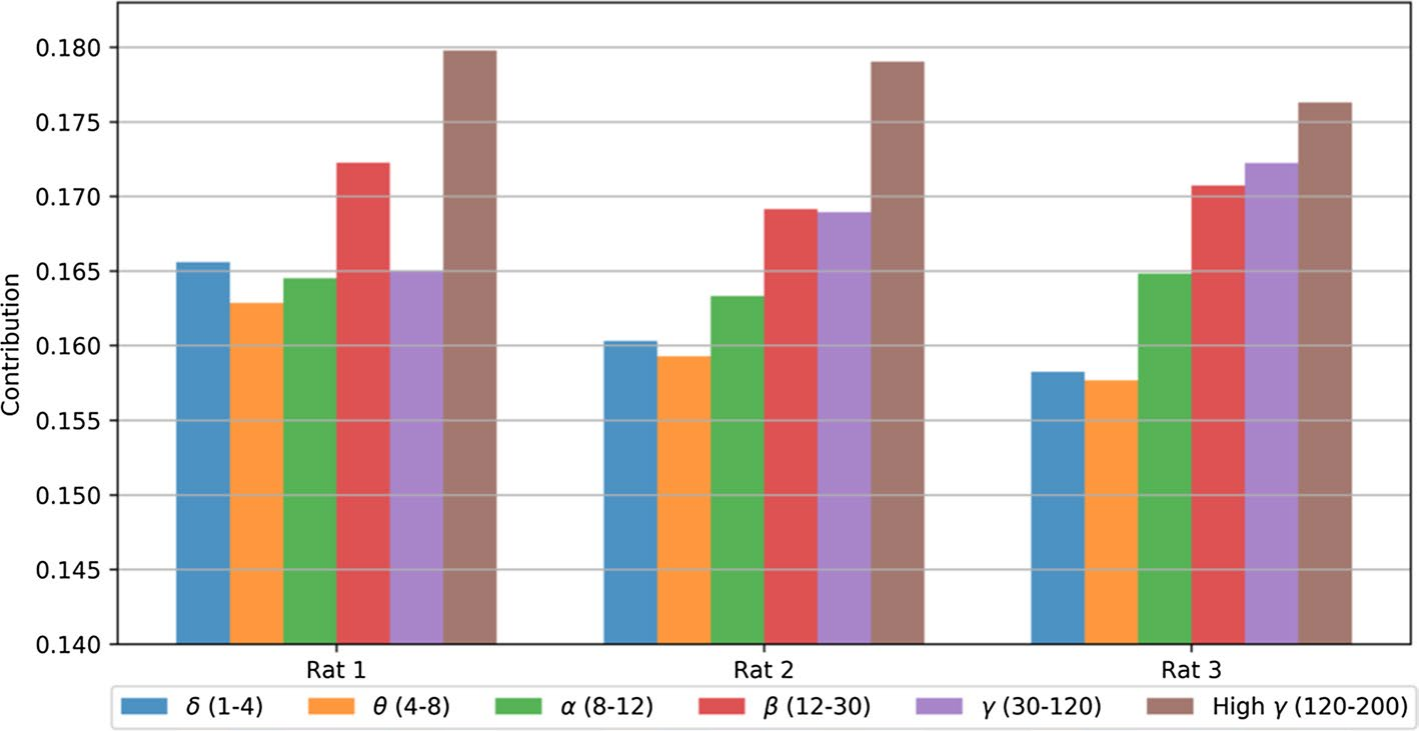
Contribution of each Frequency band in LSTM prediction. The mean value of all 7 folds is illustrated. Standard errors were negligible, therefore not shown

The contribution of each frequency band is calculated according to Eq. (16). The mean values of contributions for all the 7 folds are illustrated in Fig. 3. The standard error values were negligible; therefore, not displayed. Figure 3 shows that higher frequency bands have more contribution in predicting the force values.

### Alternative recurrent cells

**Fig. 4:**
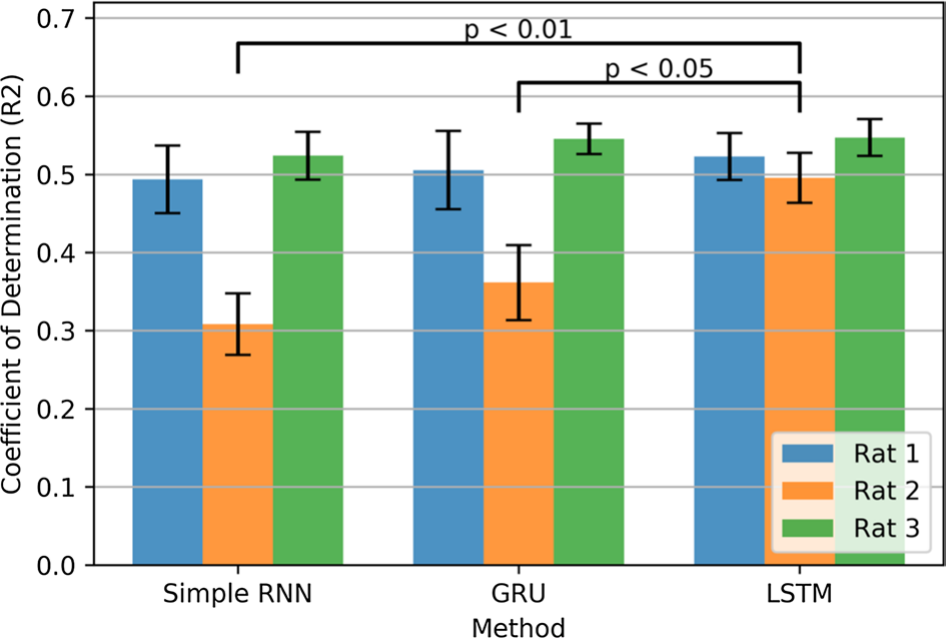
Coefficient of Determination for Networks with different types of recurrent cells. LSTM-based network significantly outperformed GRU-based and Simple RNN-based networks with (p < 0.05) and (p < 0.01) respectively

For all the above analyses, we used LSTM cells as the main recurrent block in the network. However, other recurrent cells, like Simple RNN, and GRU, are also able to remember relevant information across time sample. To compare these recurrent cells, we trained and evaluated the same network structure with three types of recurrent cell, i.e., Simple RNN, GRU, and LSTM. The LSTM-based network showed a higher Coefficient of Determination (R2) for three rats (Fig. 4). The mean (R2) value for all rats in LSTM-based network was significantly higher than GRU-based (p < 0.05), and Simple RNN-based (p < 0.01) networks. (Wilcoxon signed-rank test for three 7 folds and three rats).

### Alternative regression models

**Fig. 5:**
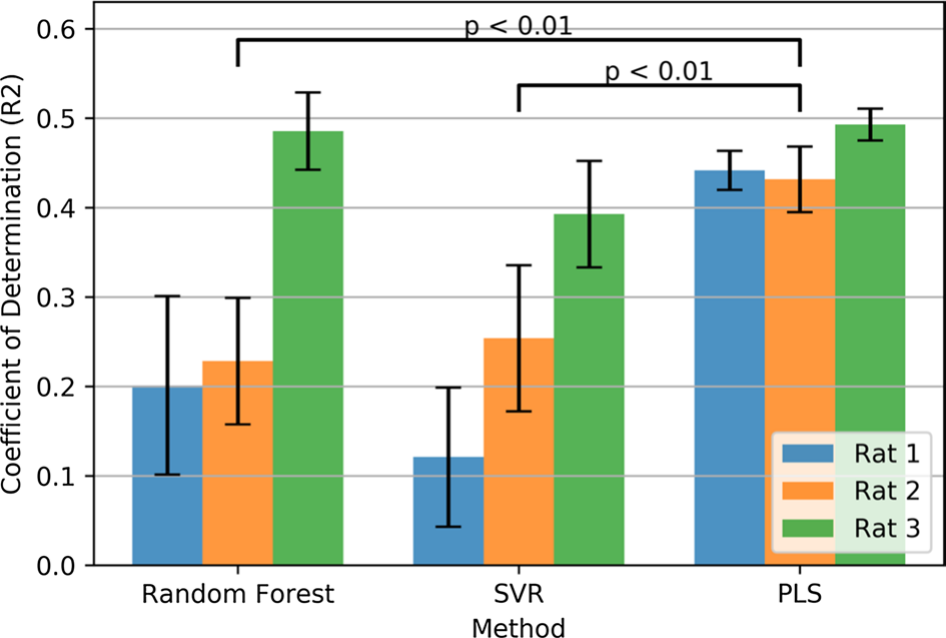
Coefficient of Determination for three regression methods. PLS regression significantly outperformed SVR and Random Forest (p < 0.01)

We used PLS as the main method of regression for comparing the proposed LSTM-based neural network. Additionally, we compared PLS results with Support Vector Regression (SVR) from the family of kernel methods, and Random Forest with bootstrapping from ensembled learning methods. Figure 5 shows the Coefficient of Determination (R2) for predicted force value by Random Forest, SVR and PLS regression, respectively. The feature extraction process, number of time lags, and validation were similar for all the method. PLS regression had higher R2 value compared to Random Forest (p < 0.01) and SVR (p < 0.01) method (Wilcoxon signed-rank test for three rats, and 7 folds).

## Discussion

### Time lag features

Using multiple time lag features is a common practice in decoding neural data. However, this time lags increase the dimensionality of the feature space dramatically. For instance, in this study, 96 features were extracted from 6 frequency bands and 16 channels. The dimensionality will increase to 960 only with 10 time lags. This increment will drastically increase the possibility of over-fitting. LSTM based networks, owing to their intrinsic potential to carry relevant information from previous samples, can recall useful information in previous LFP sample to predict the current force observation without directly providing time lags in features. Therefore, the LSTM network is less prone to over-fitting caused by a large number of features, and there is no need to optimize the number of lags in the initial feature extracting process. On the other hand, as it can be seen in Fig. 2, the decoding performance of PLS decreases with reducing the number of time lags.

### Predicted force values

As it can be seen in Fig. 1, LSTM Network can predict zero force values, but PLS prediction fluctuates around the zero values. PLS and other linear methods can only generate output values that are a linear combination of the predictors. Therefore, the nonlinear characteristics of the systems are always estimated with the closest linear model. On the other hand, neural networks, in this case, LSTM Network, has nonlinear components (11) in its structure which can model the nonlinearity in the system. Furthermore, the activation function of the output layer can be selected in a way that can improve decoding performance. For instance, in this study, Rectified Linear Unit (ReLU) was deliberately chosen as the activation function of the last layer in the LSTM network. ReLU maps all negative values to zero and acts as a simple line for positive values which makes it easier for the network to predict zero force values.

### Contribution of each frequency band

Figure 3 shows that higher frequency bands, $\beta$ (12–30 Hz), $\gamma$ (30–120 Hz) and high-$\gamma$ (120–200 Hz), had more contribution, for rat 2 and rat 3, in predicting the force values in LSTM network. This significance of higher frequency bands in neural decoding was observed in the previous study on the same data set [12].

### Alternative recurrent cells

We compared the proposed network structure with different recurrent cells. LSTM cell showed the highest decoding accuracy (Fig. 4). Both GRU and LSTM are gated structures and use a gating mechanism to remember (forget) relevant (irrelevant) information. However, higher accuracy of LSTM is probably because LSTM leverages an extra path for carrying information across the time sample. Simple RNN cell, as expected, had the lowest decoding performance because of the well-known problem of vanishing gradient through time.

### Alternative regression methods

In addition to PLS, we examined the decoding performance of two other regression methods from different families of regression techniques. Both SVR and Random Forest showed lower decoding performance compared to PLS (Fig. 5). The underperformance of SVR and Random forest, we believe, is due to a large number of features and consequently, over-fitting on the training data set. PLS, on the other hand, has an intrinsic mechanism for mitigating a large number of features without requiring a separate feature selection or dimensionality reduction.

## Conclusion

In this study, we introduced an LSTM Network, which can learn both the nonlinearity and the intrinsic dynamics of data. The overall results show that there is rich information in LFP signal for decoding fine movements like force, and the proposed network can predict this continues movement accurately, which can be used in LFP-based BCI systems.

## Methods

### Behavioral task

**Fig. 6:**
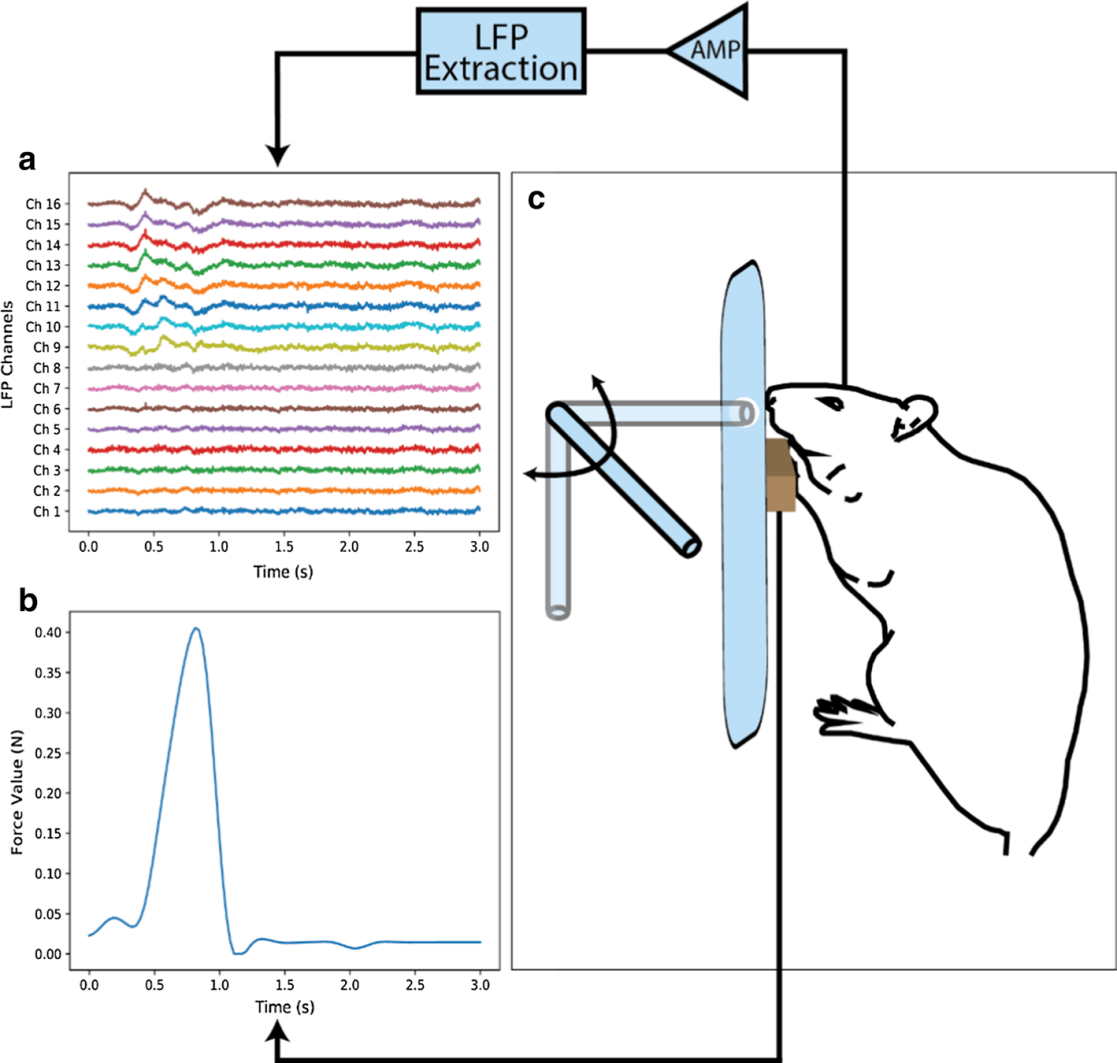
The experimental setup. **a** Raw LFP signal of 16 channels and one arbitrary trial. **b** The force profile recorded simultaneously with LPF shown in **a**. **c** The experiment setup, the force applied to the sensor was linearly mapped to the rotation of a lever. The rat was rewarded if the applied force reached 0.15 N. Neural signal, and the applied force were recorded simultaneously

In this study, three Wistar rats – average weight between 200 and 300 g—were trained to push a load cell (1 DOF) in order to receive a drop of water from a rotating lever as a reward. The applied pressure between 0 and 0.15 N was linearly mapped to 0–90 degrees of rotation in the lever. The load cell was placed 10 cm above the setup floor, and due to the negligible movement of the load cell, the position and orientation of the forelimbs were stable while performing the task (Fig. 6). If the applied force on the load cell exceeded the 0.15 N threshold, after a 1.5 s delay, the rat was rewarded. No start or end cue was defined; therefore, the timing of each trial was spontaneous.

### Structure of micro-arrays

A 4 × 4 micro-wire array with 500 μm inner wire distance was constructed using 25 μm Platinum/Iridium teflone-covered wires (Microprobes Inc., Gaithersburg, USA) with 500–800 K${\Omega }$ Impedance.

### Implantation of micro-arrays

After training, the micro-array was implanted in the primary motor cortex (M1) of three rats. The array was placed contralateral to their dominant hand. All three rats performed the task with their right hands; therefore, the arrays were placed on the left hemisphere. The surgery starts with anesthetizing the animal by administrating 100 mg/kg Ketamine and 10 mg/kg xylazine. The depth of the anesthesia was determined by toe pinching and monitoring respiration rate. Then, an incision was made in the head skin midline, and all the tissue was removed from the scalp in order to make head bone accessible. Afterward, Bregma, Lambda and the proper craniotomy positions were marked. One screw in the posterior of the lambda point and five other screws were placed to connect the ground and secure the area respectively. Then, the forelimb region of M1 was pinpointed using rat brain atlas. In the next step, the center of the micro-array was implanted 1.6 mm anterior to Bregma, 2.6 mm lateral to the midline, and 1.5 mm deep under the dura matter surface, covering all forelimb area. Finally, the area was sealed with dental acrylic. In order to avoid infection and assuage the pain, 0.2 mg/kg Meloxicam and 5 mg/kg Endrofloxacin for two days after the surgery. More detailed information on the task and surgery can be found in [12].

### Ethical considerations

All the rats belonged to the Neuroengineering and Neuroscience Research Laboratory of Iran University of Science and Technology, and the use of the animals in this study was approved and authorized by the local committee. After completion of the study, the rats were euthanized by exposure to CO_2_ and then, euthanasia was confirmed by continuing the gas exposure for 20 min after the respiratory arrest according to NIH guidelines. For more information, see Ethics approval and consent to participate section.

## Data recording

Two weeks after the surgery, the rats were placed in the task setup. Then, the neural and force data were recorded simultaneously. The implanted micro-array was connected to the preamplifier of the recording device using the implanted connector. The initial sampling rate was 10 kHz. The spikes were removed by filtering the signal between 300–3000 Hz and then manually thresholding for each channel. Then the LFP signal was extracted by filtering the signal between 0.1 and 500 Hz and downsampling to 1000 Hz. Regarding the force signal, there were negligible components above 5 Hz; therefore, the force signal was filtered and then downsampled 10 samples per second. All of the filtering processes were performed with a 4th order Butterworth filters both forward and backward. As mentioned before, the rats were free to do the task anytime; 1 s before and 2 s after the 0.15 N threshold was considered as a trial.

### Data preprocessing

The final shape of data for each trial is a matrix of 3000 by 16, representing time samples (three seconds of data with a sampling rate of 1000 samples per second) and channels respectively. There are 74, 79 and 80 successful trials for rat 1, rat 2 and rat 3 respectively.

**Fig. 7:**
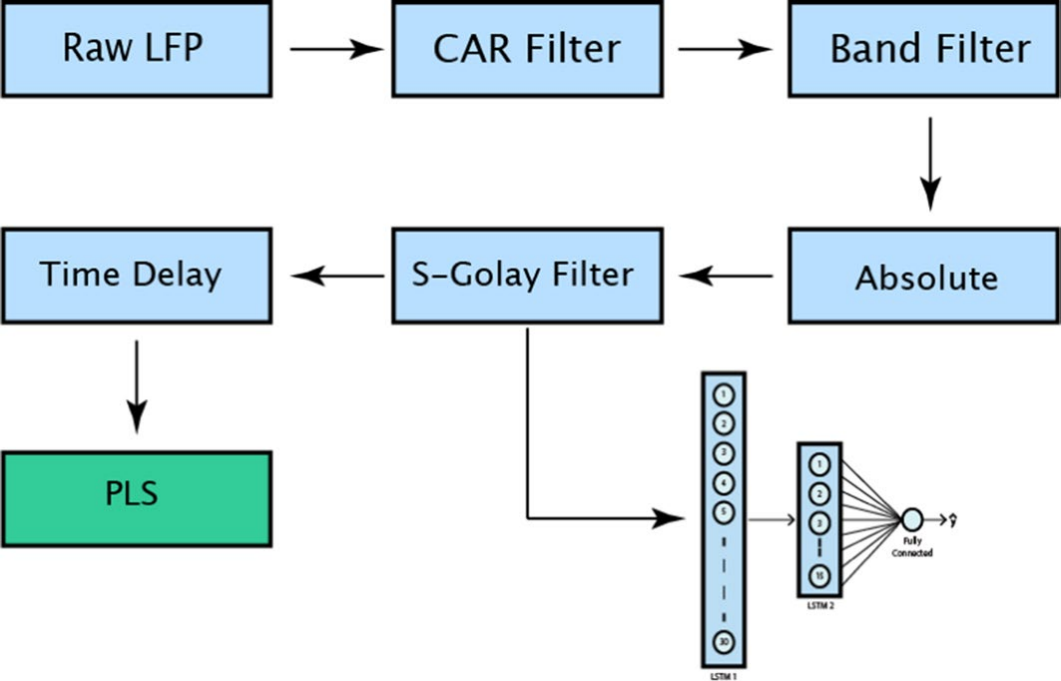
Feature extraction pipeline

The first step is to remove the noise by performing a CAR (Common Average Reference) filter on the data [26]. In using CAR, we assume that the noise is a common component existing on all the channels. Therefore, by removing the mean of all channels from each channel, the common noise can be removed. Using CAR has shown to improve the decoding performance in both methods. In the next step, the signal is decomposed into 6 frequency band. The filter band consists of $\delta$(1- 4 Hz), $\theta$(4–8 Hz), $\alpha$(8–12 Hz), $\beta$ (12–30 Hz), $\gamma$ (30–120 Hz) and high-$\gamma$ (120–200 Hz). Then, the absolute value was calculated, and the signal was smoothed with a 3rd order Savitzky–Golay filter with 150 sample window length. Using Savirzky–Golay improves the decoding mainly because it preserves the local minima in the signal. Next, the data is centered and normalized by subtracting the mean and dividing the signal by the standard deviation. Finally, the signal is downsampled in order to equalize the number of force and LFP samples. Now the dimension of feature space is (6 filter bands * 16 channels = 96) for each sample of data. In the end, for each trial, the neural data and the target force values will be matrices of size (30, 96) and (30, 1), respectively. The feature extraction pipeline is summarized in Fig. 7.

### PLS-exclusive preprocessing step

In PLS, SVR, and Random Forest algorithm, for predicting the current time sample, in addition to features for the current time sample, the features from previous time samples are included. In this study, 10 sample time lags were included in the prediction of each time sample. Therefore, the dimensionality of data will increase to (10 lags * 6 filter bands * 16 channels) 960.

### PLS mode

The general model of PLS is shown in (1) and (2). X and Y are predictor matrix and measurement vector, respectively. In this study X is a (n: number of samples, m: number of features) matrix containing features for all the time samples and y is a (n: number of samples, 1) vector for force values. The goal here is to predict y values using X.

1 $$X = TP^{T} + E$$

2 $$Y = UQ^{T} + F$$

In PLS, unlike general linear models like Least Squares, instead of working directly with X and Y, their latent variables are used. In our underlying model, T and U are two $n \times l$ matrices which are scores of X and Y respectively. P and Q are orthogonal loading matrices with the size of $m \times l$ and $p \times l$ respectively. E and F are two i.i.d Gaussian random variables.

Generally, PLS tries to explain the latent variable of Y with most variance, using the latent variable of X that describes it (it refers to the latent variable of Y) the best. Therefore, the model does not require feature selection and can reduce the possibility of over-fitting made by a large number of features, making it the ideal choice for neural data.

In PLS regression problem, the final goal is to find a weight vector $\beta$ and an intercept $\beta_{0}$ which linearly relates the predictors to the measured values as shown in (3).

3 $$y = X\beta + \beta_{0}$$

There are many variants of PLS methods and solving approaches to find loading and scoring matrices. In this study, we used PLS-1 method, which is a well-known and wildly used method for solving the PLS regression problem for cases in which Y is a vector. PLS-1 finds columns of loading matrices one by one in a stepwise manner. PLS-1 can be summarized in the following steps:

Step 1: Find an initial loading weights by finding the direction in which the covariance between X and y maximized and name it w.

4 $$w = X^{T} y$$

Step 2: Find the first score column by projecting $X$ onto $w$ and name it $t$.

5 $$t = Xw$$

Step 3: Find the first loading vectors of X and y by projecting X and y on normalized score vector $t$ found in step 2 and call them $p$ and $q,$ respectively.

6 $$\begin{aligned} p & = X^{T} \frac{t}{{t^{T} t}} \\ q & = y^{T} \frac{t}{{t^{T} t}} \\ \end{aligned}$$

Step 4: Remove all the information of the first score and loading vectors from X and y.

7 $$\begin{aligned} X_{new} & = X - tp^{T} \\ y_{new} & = y - tq^{T} \\ \end{aligned}$$

Step 5: Jump to step 1 and use $X_{new}$ and $y_{new}$ to find the next loading and score vectors. Then, iterated $l$ times for the desired number of components. Finally, concatenate all the loading, score and loading weights. All the q values calculated in step 3 are scalars; therefore, the concatenation of q values will lead to a vector Q.

8 $$\begin{aligned} W & = \left[ {w_{1} , w_{2} , \ldots , w_{l} } \right] \\ T & = \left[ {t_{1 } , t_{2 } , \ldots , t_{l} } \right] \\ P & = \left[ {p_{1} ,p_{2} , \ldots , p_{l} } \right] \\ Q & = \left[ {q_{1} ,q_{2} , \ldots , q_{l} } \right] \\ \end{aligned}$$

Step 6: Calculate regression weights $\beta$ and regression intercept $\beta_{0}$ from the calculated Matrices calculated in step 5.

9 $$\begin{aligned} \beta & = W\left( {P^{T} W} \right)^{ - 1} Q \\ \beta_{0} & = q_{1} - p_{1}^{T} \beta \\ \end{aligned}$$

In order to evaluate the performance of the PLS model, we used seven-fold cross-validation. Furthermore, The number of the latent variables are selected based on Wold’s criterion [27] shown in (10). PRESS represents the prediction error of the model when first $l$ components are used for prediction.

10 $$R_{Wold} = \frac{{PRESS\left( {l + 1} \right)}}{PRESS\left( l \right)}$$

Baibing et al. showed that using Wold’s criterion can improve the performance of the model compared to other approaches which try to find the optimal number of components [28]. To find the optimal number of components, we performed ten-fold cross-validation over train data and considered the number of components optimum when $R_{Wold}$ reached 0.9. The best number of components are six, four and five components for rat 1, rat 2 and rat 3, respectively.

## LSTM model

Inspired by classical recurrent neural networks, Long Short-Term Memory networks receive the data samples sequentially, and it uses the latest prediction for predicting the next sample of data. Classical RNNs have a feedback loop which brings back the latest outputs of the network in the input. This structure design leads to various problems such as exploding or vanishing gradient during the training of the network. To address these problems, LSTM networks share an extra parameter, cell state, between sequences that gives them the ability to remember/forget important/irrelevant features of data in any part of the sequence.

**Fig. 8:**
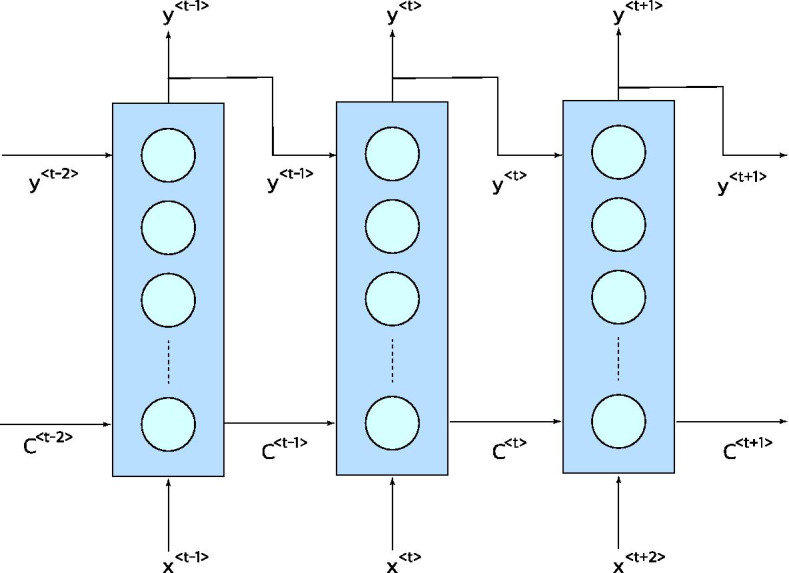
Unfolded LSTM network. Three time steps of LSTM are unfolded. In each step, LSTM receives a new input, last output and last carry and generates the next output and the next carry

LSTM network can have various input and output structure layers. For instance, LSTM can receive all the input samples and return one output at the end of receiving all input samples, or it can yield an output for each input sample. In this work, as it can be seen in Fig. 8, the network has one output for each input value in the sequence.

**Fig. 9:**
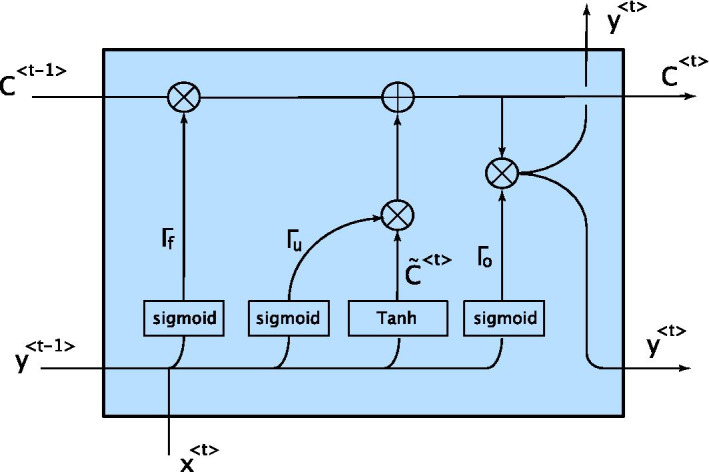
LSTM structure. Inputs, outputs and LSTM gates and their connections are illustrated

The formulation for the LSTM network can be seen in Eqs. (11), (12), (13).

11 $$\tilde{C}^{\left\langle t \right\rangle } = {\text{tanh}}\left( {W_{c} \left[ {y^{{\left\langle {t - 1} \right\rangle }} ,x^{\left\langle t \right\rangle } } \right]} \right)$$

12 $$\begin{aligned} {\Gamma }_{u} & = {\text{sigmoid}}\left( {W_{u} \left[ {y^{{\left\langle {t - 1} \right\rangle }} ,x^{\left\langle t \right\rangle } } \right]} \right) \\ {\Gamma }_{f} & = {\text{sigmoid}}\left( {W_{f} \left[ {y^{{\left\langle {t - 1} \right\rangle }} ,x^{\left\langle t \right\rangle } } \right]} \right) \\ {\Gamma }_{o} & = {\text{sigmoid}}\left( {W_{o} \left[ {y^{{\left\langle {t - 1} \right\rangle }} ,x^{\left\langle t \right\rangle } } \right]} \right) \\ \end{aligned}$$

13 $$\begin{aligned} C^{\left\langle t \right\rangle } & = {\Gamma }_{u} \odot \tilde{C}^{\left\langle t \right\rangle } + {\Gamma }_{f} \odot C^{{\left\langle {t - 1} \right\rangle }} \\ y^{\left\langle t \right\rangle } & = {\Gamma }_{o} \odot C^{\left\langle t \right\rangle } \\ \end{aligned}$$

x and y are one value of a sequence of input and output sample of data. $W_{c}$, $W_{u}$, $W_{f}$ and $W_{o}$ are carry, update, forgetting and output weights, respectively, which are going to be learned during the training process and $\odot$ stands for the element-wise product. The LSTM algorithm can be summarized in the following steps. First, using the current input sample and the previous output sample, a potential carry value, i.e., $\tilde{C}^{\left\langle t \right\rangle }$, is calculated using (11). Then again using the last output and the current input, the value of update, forget, and output gates are determined according to (12). These gates can have values between 0 and 1. For instance, in the extreme case in which ${\Gamma }_{u}$ = 1 and ${\Gamma }_{f}$ = 0, the network will fully forget the previous values and update the carry with the new carry potential value according to (13). Then, using the update and forget gates, the final carry value for the current step is calculated. Finally, the estimated output is calculated by the dot product of the current carry value and the output gate. In the original model, bias values are considered in the Eqs. (11) and (12), but in this study, the bias values were eliminated due to their negligible effect on the results and reducing the trainable parameters of the model. Furthermore, the output activation of Among different variants of LSTM structures, we used the vanilla LSTM structure because it is shown that other structures do not show a significant performance improvement in various tasks[29]. The summary of the LSTM structure is shown in Fig. 9.

### Network structure

**Fig. 10:**
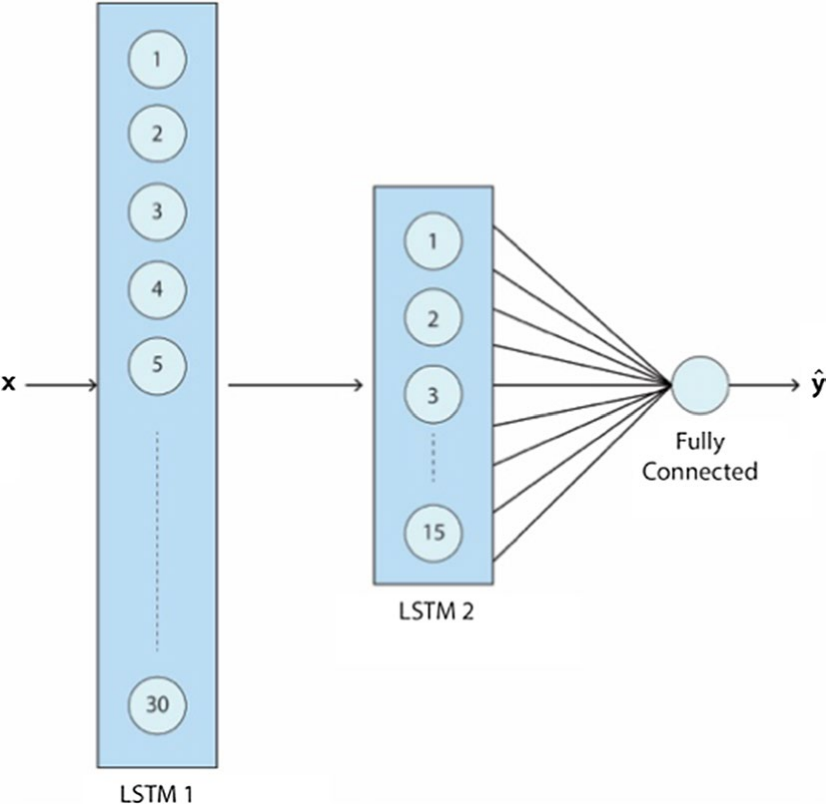
Network structure. The network consists of two LSTM layers and one fully connected neuron

The network structure used in this study is illustrated in Fig. 10. The network consists of two LSTM layers that are connected to a single fully connect neuron. The first LSTM layer has 30 units, and the overfitting in this layer is controlled by a dropout both in the forward and recurrent path. Also, the layer has no bias term. Second LSTM layer consists of 15 units with both forward and recurrent dropout. The output of the second layer is fully connected to a single neuron with ‘ReLU’ activation. The weights of the fully connected neuron are regulated with L2-norm in order to mitigate over-fitting.

The network is optimized using Adam [30] optimizer, and we set $\beta_{1} = 0.9$ and $\beta_{2} = 0.999$ as recommended in [30]. Mean absolute error was selected as cost owing to its robustness to noise. The number of epochs to train, learning rate, values for dropout rates, batch size, and regularization value of fully connected layers are hyperparameters of the network. Seven-fold cross-validation is used to evaluate the performance of the network and the PLS method. We trained the neural network with individual trials, and for PLS and other methods, training trials were concatenated. In each fold, 20% of training data is used for validation and selecting the optimum values for hyper-parameters. We used a Bayesian Optimization toolbox, Hyperopt [31], for selecting the optimum values of hyper-parameters. The Bayesian optimizer selected the optimal combination of values from the table of hyper-parameters (Table 5) that showed the best performance on validation data.

**Table 5:** Hyper-parameters of the network and their possible values

Hyper-parameter	Values
Layer 1 forward dropout	{0, 0.1, 0.2, 0.3, 0.4, 0.5}
Layer 1 backward dropout	{0, 0.1, 0.2, 0.3, 0.4, 0.5}
Layer 2 forward dropout	{0, 0.1, 0.2, 0.3, 0.4, 0.5}
Layer 2 backward dropout	{0, 0.1, 0.2, 0.3, 0.4, 0.5}
Regularization value	{0, 0.1, 0.2, 0.3, 0.4, 0.5, 0.6, 0.7, 0.8, 0.9}
Learning rate	{0.001, 0.0015, 0.002, …, 0.003}
Batch size	{5, 10, 15, 20,30}
Number of epoch	{30, 50, 70, 100, 120}

### Alternative method

In addition to PLS, we used SVR and Random Forest with bootstrapping to decode the applied force from the LFP signal. The feature extraction and validation process for SVR and Random Forest were identical to that of PLS. For SVR, we used RBF kernel with kernel coefficient $\gamma = \frac{1}{num features}$. We selected the regularization parameter (C) base on fivefold cross-validation on training data. As for Random Forest, we consider the maximum number of 100 trees and the maximum number of features for the best split was selected to be the square root of the number of features.

## Performance criteria

Coefficient of correlation (r) and coefficient of determination ($R^{2}$) were used to evaluate the performance of the models. Coefficient of correlation shows the overall resemblance between the observed and predicted values. On the other hand, the coefficient of determination can show how much of the variance in the observed data exist in the predicted values. The formulation of (r) and ($R^{2}$) are represented in (14) and (15) respectively.

14 $$r = \frac{{\mathop \sum \nolimits_{i = 1}^{n} \left( {y_{i} - \tilde{y}} \right)\left( {\hat{y}_{i} - \widetilde{{\hat{y}}}} \right)}}{{\sqrt {\mathop \sum \nolimits_{i = 1}^{n} \left( {y_{i} - \tilde{y}} \right)^{2} } \sqrt {\mathop \sum \nolimits_{i = 1}^{n} \left( {\hat{y}_{i} - \widetilde{{\hat{y}}}} \right)^{2} } }}$$

15 $$R^{2} { } = 1 - \frac{{\mathop \sum \nolimits_{i = 1}^{n} \left( {y_{i} - \hat{y}_{i} } \right)^{2} }}{{\mathop \sum \nolimits_{i = 1}^{n} \left( {y_{i} - \tilde{y}} \right)^{2} }}$$

In this formulation, $y_{i}$ is the ith sample of the target value and $\tilde{y}$ is the mean of the target value. In the same manner, $\hat{y}_{i}$ is the ith sample of predicted value and $\widetilde{{\hat{y}}}$ is the mean value of the predicted value.

### Contribution of each frequency band

The weights of the first LSTM layer contain information about the contribution of each feature of neural data for predicting the force value. Therefore, according to (16), the absolute values of weight related to each frequency band are added and then normalized by the sum of the absolute values of all the weights. The contribution value was calculated and averaged for in all validation folds.

16 $$C_{band} = \frac{{\mathop \sum \nolimits_{channel = 1}^{16} \left| {W_{band, channel} } \right|}}{{\mathop \sum \nolimits_{channel = 1}^{16} \mathop \sum \nolimits_{band = 1}^{6} \left| {W_{band, channel} } \right|}}$$

### Implementation

Neural network models were implemented in TensorFlow, using Keras API [32]. We implemented the PLS model in Python 3.8 and used python machine learning API, Scikit-learn [33] for SVR and Random Forest.

## Supplementary information

**Additional file 1:** 7-times seven-fold CV results. We present an extended version of Table 1 and Table 2. For each rat, we performed 7-times seven-fold cross validation. Correlation Coefficient (r) and Coefficient of Determination ($R^{2}$) of PLS and LSTM-based network are reported. For all rats, LSTM-based network shows significantly higher (r) and ($R^{2}$) values.

## Data Availability

The dataset analyzed in this manuscript is not publicly available. However, the data used for this study can be provided upon reasonable request. Request to access the dataset should be directed to MRD, daliri@iust.ac.ir.

## Abbreviations

BCI: Brain computer interfaces
CSP: Common spatial patterns
ECG: Electromyogram
EMG: Electromyogram
GRU: Gated recurrent unit
LFP: Local field potentials
LSTM: Long short-term memory
MNE: Minimum noise estimate
PLS: Partial least squares
ReLU: Rectified linear unit
RNN: Recurrent neural network
